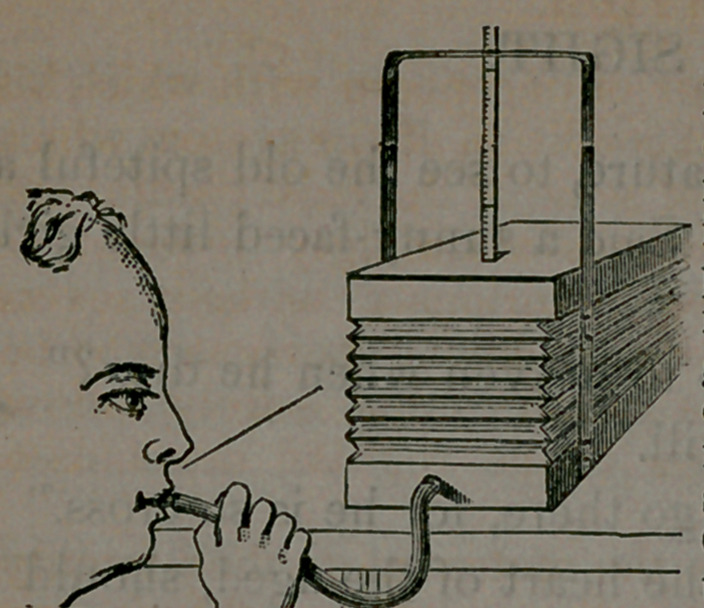# Spirometer

**Published:** 1878-03

**Authors:** 


### Dr. Hall's Portable Spirometer.

This is the only Spirometer that has ever met the requirements of purchasers. It is compact, durable, and mathematically correct; its capacity is 400 cubic inches. The wood is of polished black walnut, and all the metal parts are nickel plated. The guide and scale are hinged, so as to close down on the top of the instrument when not in use, making it small enough to be enclosed in a box 6 by 12 inches and 3 inches deep, so that it may be conveniently carried.

The Spirometer should be used where the air is pure and free from dust. It is adjusted by simply raising the guide into position.

DIRECTIONS.

Draw in the breath until the lungs are fully inflated, and then blow quickly through the rubber tube into the Spirometer until no more air will leave the lungs. The figure on the scale, which is even with the top of the guide, indicates the number of cubic inches of air expelled from the lungs. The first few trials will not be a fair test of the lung capacity particularly with persons of sedentary habits, whose lungs are seldom brought into full action, and therefore they will require considerable practice before they can fully inflate them. When used regularly and with moderation the Spirometer expands the chest, and is not only a most valuable remedy for diseased lungs, but the best means of protecting and developing lungs that are not strong.

When it is remembered that the average lung capacity of men of medium size is 200 cubic inches and that very many take into the lungs less than 50 cubic inches of air at each inspiration, leaving three quarters of the lungs in a dormant state, the importance of regular and systematic exercise for the lungs will be appreciated.

AS A TONIC

nothing equals the free introduction of pure air into the lungs, where it comes in contact with the blood, giving it that vitality upon which existence depends.

DEBILITY

always follows weak respiration, because insufficient air for the lungs, means insufficient nutrition for the whole body. Debilitated persons are benefitted by active exercise in the open air, for bodily exercise compels them to admit more air into the lungs than when they are inactive.

It is not intended that the use of the Spirometer shall take the place of such exercise but it will be found to be the best substitute for it.

SLEEPLESSNESS

The use of the Spirometer just before bed-time induces sleep, by drawing the blood from the brain to the lungs thus arresting nervous excitement, and giving rest to the weary and overworked brain.

LUNG MEASUREMENT.

Any competent physician can soon learn to estimate quite accurately the natural lung capacity of any person he sees; then by finding the actual lung capacity with the Spirometer, he can readily determine how much of the lung, if any is inactive, or diseased.

The Spirometer then, is the most important of all the means employed by physicians for accertaining the condition of the lungs.

———o———

**Price $7.00. When ordered to be sent by mail 50 cents extra must be sent to pay postage. Address, E. H. Gibbs, Editor of "Hall's Journal of Health" New York.**

## Figures and Tables

**Figure f1:**